# Psychometric properties and clinical utility of the Scale for Suicidal Ideation (SSI) in adolescents

**DOI:** 10.1186/1471-244X-5-8

**Published:** 2005-02-03

**Authors:** Matti M Holi, Mirjami Pelkonen, Linnea Karlsson, Olli Kiviruusu, Titta Ruuttu, Hannele Heilä, Virpi Tuisku, Mauri Marttunen

**Affiliations:** 1 Department of Mental Health and Alcohol Research, National Public Health Institute, Helsinki, Finland; 2 Department of Psychiatry, Lapinlahti Hospital, Helsinki University Central Hospital, Helsinki, Finland; 3 Department of Adolescent Psychiatry, Peijas Hospital, Helsinki University Central Hospital, Helsinki, Finland; 4 Department of Psychiatry, Kuopio University and Kuopio University Hospital, Kuopio, Finland; 5 Department of Psychiatry, Turku University Central Hospital, Turku, Finland

## Abstract

**Background:**

Accurate assessment of suicidality is of major importance in both clinical and research settings. The Scale for Suicidal Ideation (SSI) is a well-established clinician-rating scale but its suitability to adolescents has not been studied. The aim of this study was to evaluate the reliability and validity, and to test an appropriate cutoff threshold for the SSI in a depressed adolescent outpatient population and controls.

**Methods:**

218 adolescent psychiatric outpatient clinic patients suffering from depressive disorders and 200 age- and sex-matched school-attending controls were evaluated by the SSI for presence and severity of suicidal ideation. Internal consistency, discriminative-, concurrent-, and construct validity as well as the screening properties of the SSI were evaluated.

**Results:**

Cronbach's α for the whole SSI was 0.95. The SSI total score differentiated patients and controls, and increased statistically significantly in classes with increasing severity of suicidality derived from the suicidality items of the K-SADS-PL diagnostic interview. Varimax-rotated principal component analysis of the SSI items yielded three theoretically coherent factors suggesting construct validity. Area under the receiver operating characteristic (ROC) curve was 0.84 for the whole sample and 0.80 for the patient sample. The optimal cutoff threshold for the SSI total score was 3/4 yielding sensitivity of 75% and specificity of 88.9% in this population.

**Conclusions:**

SSI appears to be a reliable and a valid measure of suicidal ideation for depressed adolescents.

## Background

Accurate assessment of suicidality is of major importance in both clinical and research settings. Adolescent suicide occurs usually in the context of an active, often treatable, but unrecognized or untreated mental illness [1-3]. The increase in the antidepressant treatment of adolescents in the USA [4] may partly explain the decline in the incidence of youthful suicide [5], though recently some reports have connected SSRI-treatment in adolescents to an increase in suicidality [6].

Suicide attempts are complex acts for which no single set of clinical features can be expected to be a good predictor [7]. Suicidal ideation, self-harming, suicide attempts and completed suicides are different forms of suicidality. Although the domain of suicidal behavior probably is multidimensional [8], a continuum from suicide ideation to suicide attempts has been reported in youthful clinical populations [9,10]. Thus, although most patients with suicidal ideation do not attempt suicide, identification and assessment of severity of suicidal ideation is of major importance.

The Scale for Suicidal Ideation (SSI) [11] was designed to measure the intensity, pervasiveness, and characteristics of suicidal ideation in adults. It also aims to assess the risk of later suicide attempt in individuals who have thoughts, plans, and wishes to commit suicide [12]. It is a well-established clinician-rating scale and is presented in a semi-structured interview format.

The psychometric properties of the SSI have been evaluated in adult population and in inpatient children. Both in a sample of adult psychiatric inpatients and in a sample of inpatient children the internal consistency of the scale was good [11,13]. SSI reportedly has three dimensions [11], which have been only partly replicated in some factor analytical studies [e.g. [13,14]]. SSI has been found to converge with scales measuring related constructs e.g. hopelessness and depression in adults, and hopelessness, depression and self-harm in children [11,13].

The predictive validity of the SSI has been studied in a sample of hospitalised patients, where the SSI scores of those who committed suicide were not significantly higher than the scores of inpatients that did not [15]. In a sample of 3701 adult outpatients those who scored over a SSI threshold value had 5.42 times higher odds of committing suicide than those who scored under [16]. The threshold value was derived from a receiver operating characteristic (ROC) analysis that yielded optimal threshold of 1/2 for predicting future suicide. In the same study, SSI-scores inquiring the worst point in life (SSI-W) yielded an odds ratio of 13.84 for predicting suicide. A recent study that inquired retrospectively records of suicide victims to find communications that fit the SSI-items found no suicide-predicting power for the instrument [17].

Some instruments have evolved from the SSI, for example the Modified scale for suicidal ideation (MSSI) [18] that was designed to suit paraprofessionals and the Beck scale for suicidal ideation (BSS) [19] that is a self-report scale.

The SSI has been used widely in adult psychiatric populations [e.g. [20,21]], but its psychometric properties have not been evaluated in adolescents. According to a recent comprehensive review "despite its potential utility, the SSI's suitability to adolescents... remains to be elucidated" [22]. Rating scales should be validated in each patient population in which they are used. The aim of this study was to evaluate the reliability and validity of the SSI and test an appropriate cutoff threshold for clinically significant suicidal ideation in an adolescent population.

## Methods

### Sample

The study population consisted of two samples; a psychiatric outpatient sample of 218, and an age- and sex-matched control sample of 200 school-attending adolescents. The outpatients suffered from depressive mood disorder, were of ages 13 through 19, and took part in the Adolescent Depression Study (ADS). They were recruited between 1.2.1998 and 31.12.2001 from a consecutive sample of patients attending the outpatient clinics of the Department of Adolescent Psychiatry of Peijas Medical Health Care District covering approximately 210,000 inhabitants and comprising the cities of Vantaa and Kerava in the Helsinki metropolitan area, southern Finland.

Of the eligible (appropriate age, knowledge of Finnish language and adequate cognitive capacity) 660 outpatients, 624 (94.5%) were screened during their first consultation visit by the Beck Depression Inventory (BDI) [23] and the General Health Questionnaire-36 (GHQ-36) [24,25]. Those 373 (59.8%) with scores of 10 or more and 5 or more, respectively, were considered screen positives, and were asked to participate in the study. 118 (31.6%) outpatients refused and 34 (9.1%) dropped out at this stage. 221 (33.5%) remaining outpatients were evaluated by a diagnostic interview (K-SADS-PL) [26] and those 218 (33.0%) with a current depressive mood disorder were included in the study.

The control sample was drawn from the enrollment lists in four schools in the corresponding geographical area. It was a random sample of age- and sex-matched students equating the distribution of the educational level of the outpatients.

### Instruments

1) The Scale for Suicide Ideation (SSI) is a clinician-rating scale and is presented in a semi-structured interview format [11]. It consists of 19 items that evaluate three dimensions of suicide ideation: active suicidal desire, specific plans for suicide, and passive suicidal desire. Each item is rated on a 3-point scale from 0 to 2. The higher the total score, the greater the severity of suicide ideation. In some previous studies on adult suicidality a score of 6 or more has been used as a cutoff threshold for clinically significant suicidal ideation [e.g. [20]]. The psychometric properties of the SSI have been evaluated for adult psychiatric patient population; the internal consistency of the scale was found to be good (α = 0.89), and factor analysis yielded the three above-mentioned dimensions [11]. Among inpatient children rated by trained raters the factors could not be replicated; only two factors ("active suicidal desire" and a mixture of "active and passive desire") existed with miscellaneous items left over [13]. Nine trained raters did the SSI rating in our study.

2) The Schedule for Affective Disorders and Schizophrenia for School-Aged Children-Present and Lifetime (K-SADS-PL) [26] is a widely used semi-structured diagnostic interview. Suicidal behavior was determined using four questions from the screening-section of the K-SADS-PL diagnostic interview: item-1 suicidal thoughts ("1" = none, "2" = occasional, "3" = frequent), item-2 suicide attempts and their seriousness ("1" = none, "2" = ambivalent, "3" = serious) and item-3 suicide attempts and their lethality ("1" = none, "2" = not life-threatening, "3" = life-threatening). Self-harming behavior was asked using item-4, the question on deliberate self-harm without intent to die ("1" = none, "2" = occasional, "3" = frequent) in the screening section of the K-SADS-PL.

The K-SADS-PL is considered internationally reliable and valid diagnostic instrument for adolescent population [27]. It has been translated (and back translated) into Finnish and used widely in studies concerning suicidality [e.g. [9,28]]. Nine trained raters did the rating. Inter-rater reliability, assessed using 15 randomly selected videotaped interviews, was good for mood disorder diagnoses [weighted kappa [29] for MDD, other mood disorder, no mood disorder 0.87 (95 % CI 0.81, 0.93)].

3) Clinical suicidality assessment (CSA): A three-point mutually exclusive grouping of suicidality (1-non-suicidal, 2-suicide ideation, 3-suicide attempts) is a simplified version of the 5-item "Spectrum of Suicidal Behavior Scale" [30]. It has been used in both research and clinical purposes [e.g. [10]]. The grouping is done by a clinician, and is based on two simple questions "Have you thought of killing yourself?" and "Have you attempted suicide?" and on patient records when appropriate. There is some evidence supporting the predictive validity of this grouping [10] but it has not been validated by comparing it with more structured measures like the K-SADS-PL. In this study, after a brief training the treating clinicians of the outpatient clinic did the CSA. They were instructed to include in class-3 also self-mutilation and other self-harming behavior with no explicit suicide intent.

### Procedure

After a description of the study, a written informed consent was obtained from the subjects. For subjects less than 18 years consent was also asked from the parents or other legal guardians. For the community sample the K-SADS-PL and the SSI were performed at the same day by an expert clinician. For the outpatient sample the K-SADS-PL was performed within variable time from the SSI rating. The CSA was performed for the patient sample by clinicians during the beginning of the treatment.

### Statistical analysis

Central tendencies of some data were reported using medians and quartiles because of non-normal distribution. Mann-Whitney U test was used to assess the significance of differences between the two samples.

Internal consistency of the SSI was evaluated by calculation of Cronbach's α for the whole scale.

Concurrent validity of the instrument was examined by comparing it with the K-SADS-PL with the CSA classifications. SSI total scores were first assessed in 5 classes of increasing suicidality derived from the K-SADS-PL responses in the following way: 1-no suicidal ideation or acts, 2-mild suicidal ideation (score 2 on item-1), 3-severe suicidal ideation (score 3 on item-1), 4-mild suicidal acts (score 2 on any of items 2–4 regardless of ideation), 5-severe suicidal acts (score of 3 on any of items 2–4 regardless of ideation).

Then the SSI total scores were measured in 3 classes of increasing suicidal ideation severity, regardless of possible suicidal acts, derived from the K-SADS-PL responses on item 1: 1-no ideation, 2-mild ideation, and 3-severe ideation. Severe ideation (score 3) in this item was considered as "clinically significant suicidal ideation".

Finally the SSI total score was assessed in the three classes of the CSA: 1-no suicidality, 2-suicidal ideation, 3-suicidal or self-harming acts.

The statistical significance of the between-class differences was evaluated by Kruskal-Wallis test. For the analyses of concurrent validity only SSI-measurements in a range of 30 days from the K-SADS-PL and the CSA were included.

Construct validity was measured by performing a principal component analysis (PCA) with varimax rotation in the outpatient sample. The internal consistencies (Cronbach's α) of the extracted components as well as the originally reported factors [11] were calculated.

ROC-analysis was performed to evaluate the screening properties of the SSI, and the cutoff threshold for the instrument was defined by optimal trade-off between sensitivity and specificity (Youden's index [31]). The K-SADS-PL (score 3 in item-1) was used as the standard to define cases with clinically significant suicidal ideation.

SPSS 11.0 (Chicago, Illinois 60606, SPSS Inc) was used for the statistical analysis.

## Results

Eighteen percent (n = 40) of the outpatient sample were boys and 82% (n = 178) girls, in the community sample the percentages were 18.6% (n = 37) and 81.4% (n = 162), respectively. The subjects' mean age was 16.4 (SD 1.6) in the outpatient sample and 16.5 (SD 1.6) in the community sample. The median SSI total score for the patient sample was 0 (Q1–3 = 0–6) and for the community sample 0 (Q1–3 = 0-0) (z = -9.6, p = 0.000). The median SSI total score for subjects aged 13–15 was 0 (Q1–3 = 0–1) and those aged 16–19 0 (Q1–3 = 0–1) (z = -0.685, p = 0.493). The median time distance between SSI and K-SADS-PL was 21.5 days (Q1–3 = 9–36) for the patient sample and 0 days (Q1–3 = 0-0) for the control sample (z = -18.0, p = 0.000). The median time distance between SSI and the CSA was 6 days (range 0–35).

Forty-seven (21.6%) outpatients and one (0,5%) control subject had current clinically significant suicidal ideation (p = 0.000) according to the K-SADS-PL.

### Reliability

Cronbach's α was 0.95 for the whole sample, 0.81 for the community sample and 0.95 for the outpatient sample.

### Concurrent validity

146 (67%) of the outpatients and 199 (99.5%) of the controls were included in the analyses for concurrent validity, as their measurements were within the required range of 30 days.

The median SSI sum scores in the five suicidality classes derived from the K-SADS-PL were class-1 = 0 (Q1–3 = 0-0); class-2 = 5.5 (Q1–3 = 0–8); class-3 = 13 (Q1–3 = 0–18.5); class-4 = 4 (Q1–3 = 0–17.3); class-5 = 8 (Q1–3 = 0–13). The differences were significant (χ^2 ^= 111.6, df 4, p = 0.000).

The median SSI sum scores in the three classes of suicidal ideation derived from the K-SADS-PL were class-1 = 0 (Q1–3 = 0-0); class-2 = 4 (Q1–3 = 0–8); class-3 = 13 (Q1–3 = 4–18). The differences were significant (χ^2 ^= 132.6, df 2, p = 0.000).

The median SSI sum scores in the three clinical suicidality evaluation classes (only the outpatient sample) were class-1 = 0 (Q1–3 = 0–1); class-2 = 10 (Q1–3 = 5–18); and class.3 = 15 (Q1–3 = 13.3–16.6). The differences were significant (χ^2 ^= 57.9, df 2, p = 0.000).

### Construct validity

Principal Component analysis could be performed only for the outpatient sample due to a small variance of responses in the community sample. The analysis yielded a strong first unrotated factor, which explained 53% of the variance, and two more factors with eigen value > 1. The three factors and their internal consistencies after varimax rotation are presented in Table 1. The internal consistencies (Cronbach's α) of the originally reported [11] three dimensions were "active suicidal desire" α = 0.92, "preparation" α = 0.69, "passive suicidal desire" α = 0.79.

**Table 1:** Factor loadings and internal consistencies of the varimax rotated Principal Component Analysis (PCA) of the SSI in an outpatient sample of 218 adolescent outpatients with mood disorder. (* = Items that loaded identically to Beck's [11] original study) In the original study items 8, 10, 11 loaded on "active suicidal desire"-factor; items 13 and 15 on "preparation"-factor; and items 14 and 18 on "passive suicidal desire"-factor; item 17 did not load adequately on any of the factors.

**Item**		**Loadings**	
	Factor 1:	Factor 2:	Factor 3:
	(active suicidal desire)	(passive suicidal desire)	(preparation)
7. time dimension: frequency	0.824 *	0.208	0.180
6. time dimension: duration	0.764 *	0.321	0.225
4. desire to make active suicide attempt	0.753 *	0.389	0.142
9. control over suicidal action	0.746 *	0.140	0.140
1. wish to live	0.716 *	0.170	0.063
12. method: specificity/planning	0.714 *	0.383	0.303
2. wish to die	0.702 *	0.360	0.137
3. reasons for living/dying	0.689 *	0.364	0.219
14. sence of "capability"	**0.657**	0.405	0.322
13. method: availability/opportunity	**0.649**	0.409	0.285
	α = **0.94**		
			
5. passive suicidal desire	0.256	0.720 *	0.016
19. deception/concealment of suicide	0.196	0.711 *	0.201
8. attitude toward ideation/wish	0.508	**0.650**	0.177
10. deterrents to active attempt	0.242	**0.633**	0.389
11. reason for contemplated attempt	0.527	**0.619**	0.058
15. expectancy/anticipation of event	0.445	**0.603**	0.226
		α = **0.85**	
			
18. final acts	0.098	0.151	**0.802**
17. suicide note	0.358	0.001	**0.787**
16. actual preparation	0.133	0.319	0.646 *
			α = **0.65**

### Validity as a screening instrument

**Figure 1:**
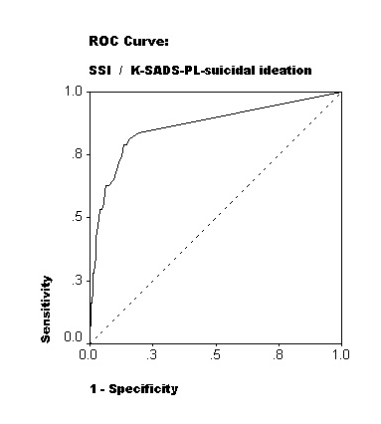
Detection of suicidal ideation by the Scale for suicidal ideation (SSI) against the K-SADS-PL as a standard, at a sample of 146 depressed adolescent outpatients and 199 age- and sex-matched controls. ROC-curve with a reference line.

ROC analysis (Fig. 1) of the SSI total score against the K-SADS-PL-confirmed suicidal ideation yielded an area-under-curve (AUC) of 0.84 for the whole sample (n = 418) and an AUC of 0.80 for the patient sample (n = 218). The optimal trade-off between sensitivity and specificity (Youden's index) was achieved at a cutoff threshold score of four or more in the whole sample as well as the patient sample. In the whole sample the sensitivity and the specificity at this threshold were 75% and 88.9%, respectively (Table 2) with 53 subjects classified incorrectly. In the patient sample the sensitivity and the specificity at this optimal threshold were 76.6% and 77.2%, respectively (Table 3) with 50 subjects classified incorrectly.

**Table 2:** Validity coefficients of different SSI cutoffs against K-SADS-PL diagnosed significant suicidal ideation at a mixed adolescent sample of 146 outpatients and 199 community controls

SSI cutoff	0–1	1–2	2–3	**3–4**	4–5	5–6	6–7	7–8	8–9	9–10	10–11
Sensitivity	77.1%	75%	75%	**75%**	66.7%	64.6%	58.3%	58.3%	58.3%	56.3%	50.0%
Specificity	83.0%	86.5%	87.6%	**88.9%**	90.0%	91.6%	93.0%	94.6%	95.9%	96.2%	96.8%
Youden	0.60	0.62	0.63	**0.64**	0.57	0.57	0.51	0.53	0.54	0.53	0.47

**Table 3:** Validity coefficients of different SSI cutoffs against K-SADS-PL diagnosed significant suicidal ideation at an adolescent sample of 146 outpatients

SSI cutoff	0–1	1–2	2–3	**3–4**	4–5	5–6	6–7	7–8	8–9	9–10	10–11
Sensitivity	78.7%	76.6%	76.6%	**76.6%**	68.1%	66.0%	59.6%	59.6%	59.6%	57.4%	51.1%
Specificity	66.7%	73.1%	74.3%	**77.2%**	78.9%	82.5%	85.4%	88.9%	91.2%	91.8%	93.0%
Youden	0.46	0.50	0.51	**0.54**	0.47	0.49	0.45	0.49	0.51	0.49	0.44

## Discussion

This study was the first to evaluate the psychometric properties of the SSI in an adolescent population. It was a part of the ongoing Adolescent Depression Study (ADS) and the sample of patients was large compared to earlier similar studies in adult populations, and probably representative of adolescent psychiatric outpatients with depressive disorders. The main finding was that the SSI appeared to be a reliable and valid instrument for evaluation of suicidal ideation in a depressed adolescent population. Its internal consistency and different aspects of validity were good and similar to what has been reported among adults.

The construct validity of the SSI was checked by Principal Component Analysis, which yielded 3 theoretically meaningful and coherent factors, only slightly different from the original ones, with good internal consistencies. This suggests good construct validity. The first factor ("active suicidal desire") was nearly identical to Beck's original one [11]. The second factor ("passive suicidal desire") included theoretically coherent items, two of which were identical to Beck's original factor of similar content. The third factor was also theoretically meaningful, included three items concerning final preparations, and had one item in common with Beck's original "preparations" factor.

The SSI converged theoretically meaningfully with both the three-class K-SADS-PL suicidal ideation-item and the clinical suicidality assessment (CSA); growing SSI scores were found within categories with increasing severity of suicidality. As to the convergence with the 5-class K-SADS-PL suicidality instrument, the results were more complex. The Kruskal-Wallis test yielded significant differences between the SSI scores in the different categories as expected, but the SSI-scores in the K-SADS-PL classes 4 and 5, with the supposedly most severe suicidality were not higher than in class 3. Classes 4 and 5 inquire about suicidal acts, and may represent a partly separate domain from suicidal ideation, which may be related to the presence of comorbid personality traits or conduct disorders. The SSI was designed to tap suicidal ideation and it may not satisfactorily tap features related with suicidal acts. In accordance with the theory of multidimensional nature of suicidality [8], severe suicidal ideation may not always be a prerequisite for suicidal acts in adolescents.

The authors are not aware of previous empirical estimations of clinically relevant cutoff for the SSI in adolescents. In this study ROC analysis of the whole sample yielded a reasonable result, but the validity coefficients for the different cutoffs of the SSI were somewhat difficult to interpret. In the community sample, there was only one subject with K-SADS-PL-diagnosed clinically significant suicidal ideation, which may have biased the analyses made with the whole sample. The results for both the whole sample and the patient sample suggest that a cutoff threshold score of four or more might be optimal for adolescents. Depending on the purpose the SSI is used, however, the emphasis between sensitivity and specificity may change, and a different threshold may be useful. For example, if the purpose is to detect the maximum number of potential suicides the cutoff threshold should be lowered to minimize the number of false negatives.

### Limitations

Several methodological limitations should be noted, some suggesting caution in interpreting the findings. Inter-rater and test-retest reliabilities, which would have given a complete picture of the reliability of the SSI, could not be evaluated in our setting; they would have required repeated SSI measurements for each subject. However, the alpha-coefficients are a marker of internal consistency, which is one indicator of reliability.

Although large and representative, the sample was a pure outpatient sample with age- and sex-matched controls, and females were over-represented. The absence of inpatients may have caused us to see the spectrum of suicidal ideation narrower than in real clinical situations. The sample was limited to an urban area in southern Finland, the generalizability of our findings to rural areas, or to other countries, is not known.

The use of K-SADS-PL as a standard for clinically significant suicidal ideation and behavior may be criticized, as the authors are not aware of a data on its validity. It is used, however, as one of the best available standards in adolescent mood disorder diagnostics, and taps suicidality with 4 relevant items.

The same rater rated the K-SADS-PL and the SSI, which is a weaker test of concurrent validity than correlating measures rated by separate raters.

### Clinical implications

The SSI can safely be used to evaluate suicidal ideation in adolescents where it seems to perform as well as in adults, where it is considered to be well established. When screening clinically significant suicidality in adolescents, a total score threshold of 3/4 may be useful.

Suicidal acts may occur among adolescents with only "mild" suicidal ideation. Thus, prevention of suicidal acts cannot rely solely on the SSI, which does not seem to tap them accurately. Furthermore, questionnaires should be only an adjunct to the clinical evaluation of suicidality.

## Conclusions

SSI appears to be a reliable and a valid measure of suicidal ideation at depressed adolescents, with a cutoff threshold value of four or more of total SSI score being an appropriate for detecting significant suicidal ideation.

## Competing interests

The author(s) declare that they have no competing interests.

## Authors' contributions

MMH analyzed the data and wrote the paper. MP interviewed patients, participated in planning the study and analyses, and writing the paper. LK, TR, HH and VT participated in planning the study, interviewed patients, and commented on the manuscript. OK participated in planning the study and the analyses, and commented on the manuscript. MM supervised the study, interviewed patients and participated in planning of the study and analyses, and writing the paper. All authors read and approved the final manuscript.

## Pre-publication history

The pre-publication history for this paper can be accessed here:


